# *Strongyloides stercoralis *hyperinfection in a patient with rheumatoid arthritis and bronchial asthma: a case report

**DOI:** 10.1186/1476-0711-9-27

**Published:** 2010-09-20

**Authors:** Levent Altintop, Burcu Cakar, Murat Hokelek, Ahmet Bektas, Levent Yildiz, Muge Karaoglanoglu

**Affiliations:** 1 Department of Internal Medicine, Ondokuz Mayis University Medical School, Samsun, Turkey; 2 Microbiology, Ondokuz Mayis University Medical School, Samsun, Turkey; 3 Pathology, Ondokuz Mayis University Medical School, Samsun, Turkey

## Abstract

**Objective:**

*Strongyloides stercoralis *is a soil-transmitted intestinal nematode that has been estimated to infect at least 60 million people, especially in tropical and subtropical regions. Strongyloides infection has been described in immunosupressed patients with lymphoma, rheumatoid arthritis, diabetes mellitus etc. Our case who has rheumatoid arthritis (RA) and bronchial asthma was treated with low dose steroids and methotrexate.

**Methods:**

A 68 year old woman has bronchial asthma for 55 years and also diagnosed RA 7 years ago. She received immunusupressive agents including methotrexate and steroids. On admission at hospital, she was on deflazacort 5 mg/day and methotrexate 15 mg/week. On her physical examination, she was afebrile, had rhonchi and mild epigastric tenderness. She had joint deformities at metacarpophalengeal joints and phalanges but no active arthritis finding.

**Results:**

Oesophagogastroduodenoscopy was performed and it showed hemorrhagic focus at bulbus. Gastric biopsy obtained and showed evidence of *S.Stercoralis *infection. Stool and sputum parasitological examinations were also all positive for *S.stercoralis *larvae. Chest radiography result had no pathologic finding. Albendazole 400 mg/day was started for 23 days. After the ivermectin was retrieved, patient was treated with oral ivermectin 200 μg once a day for 3 days. On her outpatient control at 15th day, stool and sputum samples were all negative for parasites.

**Conclusion:**

*S.stercoralis *may cause mortal diseases in patients. Immunosupression frequently causes disseminated infections. Many infected patients are completely asymptomatic. Although it is important to detect latent *S. stercoralis *infections before administering chemotherapy or before the onset of immunosuppression in patients at risk, a specific and sensitive diagnostic test is lacking. In immunosupressed patients, to detect *S.stercoralis *might help to have the patient survived and constitute the exact therapy.

## Introduction

*Strongyloides stercoralis *infects 30 - 100 million people in 70 countries in tropical and subtropical areas [1-3]. Infection usually results in asymptomatic chronic disease of the gut, which can remain undetected for decades. However, in patients receiving long-term corticosteroid therapy, hyperinfection can occur, resulting in high mortality rates (up to 87%)[1].

*Strongyloides stercoralis *is an intestinal nematode parasite. It has the ability to reproduce itself in human. Humans become infected by filariform larvae [2,4,5]. It is transmitted from the soil and penetrates into the skin. After exposure, it can migrate to the respiratory system via bloodstream. The parasite is, then, swallowed and entered the gastrointestinal tract. It penetrates the duodenum wall. The female leave eggs to small bowel mucosa. Rhabditiform larvae hatch from the eggs and expelled in feces; and then, asexual cycle developed in soil. Rhabditiform larvae turn into males and females, pass eggs to the soil; filariform larvae are developed and infected human. A short cycle also takes place in human body. Rhabditiform larvae mature and penetrate the skin in perianal area and autoinfection developed [6].

The uncomplicated intestinal form of disease produces nonspecific abdominal symptoms with or without mild sporadic diarrhea. Many infected patients are completely asymptomatic [3]. The penetration of the colon or the anal skin by filariform larvae, and its migration through lung allow reinfection of the same host.

Acute infection can lead a dermatologic sign at the larval penetration region of the skin[2]. Pulmonary symptoms including dyspnea, cough, rhonchi, wheezing occur afterwards. When the parasites are swallowed, gastrointestinal (GI) symptoms develop before the stool samples are positive for larvae. Gastrointestinal and pulmonary symptoms are non-specific and include abdominal pain, diarrhea, vomiting, adynamic ileus, small bowel obstruction and protein-losing enteropathy, pneumonia [2].

Strongyloidiasis is difficult to diagnose because the parasite load is low and the larval output is irregular. Results of a single stool examination by use of conventional techniques fail to detect larvae in up to 70% of the cases [1].

Autoinfection is the major characteristic that seperate *S.stercoralis *from the other parasite forms. *S.stercoralis *may remain dormant in human body for years. Immunosupression can trigger replication and cause fatal diseases.

Strongyloides infection has been described in immunosupressed patients with lymphoma, rheumatoid arthritis, diabetes mellitus etc.

Here we present a case with RA and bronchial asthma who was treated with low dose steroids and methotrexate.

## Case Report

A 68 years old woman was admitted to hospital with weakness, dyspepsia and cough in Samsun, Turkey. Her previous medical history was indicating that she has had bronchial asthma for 55 years and received short interval prednisolone therapy for exacerbations. She had also diagnosed RA 7 years ago and received immunusupressive agents including methotrexate and steroids at different doses for disease control. On admission to the hospital, she was on deflazacort 5 mg/day and methotrexate 15 mg/week. She had also recurrent inpatient clinic follow-up history because of resistant hyponatremia with unknown origin and moderate anemia. On her physical examination, she was afebrile, had rhonchi and mild epigastric tenderness. She had continuous nausea and she was sometimes vomiting. She had joint deformities at metacarpophalengeal joints and phalanges; but no active arthritis finding. Laboratory data revealed normochrom normocytic anemia, mild hyponatremia (Na:128 mEq). Abdomen and chest radiography were normal.

Kazuto K at all. studied in endoscopic and histopathological hyperinfection of *Strongyloides stercoralis *on the duodenum. Twenty-four (96%) of the patients, investigated were under immunocompromised condition. The abnormal endoscopic findings, mainly edematous mucosa, white villi and erythematous mucosa were observed in 23 (92%) patients. The degree of duodenitis including villous atrophy/destruction and inflammatory cell infiltration corresponded to the severity of the endoscopic findings. The histopathologic yield for identifying larvae was 71.4% by duodenal biopsy. The endoscopic findings of duodenitis were more severe in patients whose biopsies were positive for larvae than those whose biopsies were negative [2]. Those patients gastrointestinal and pulmonary symptoms are common but non-specific, and include abdominal pain, diarrhea, vomiting, adynamic ileus, small bowel obstruction (SBO) and protein-losing enteropathy, as well as pneumonia [2].

**Figure 1:**
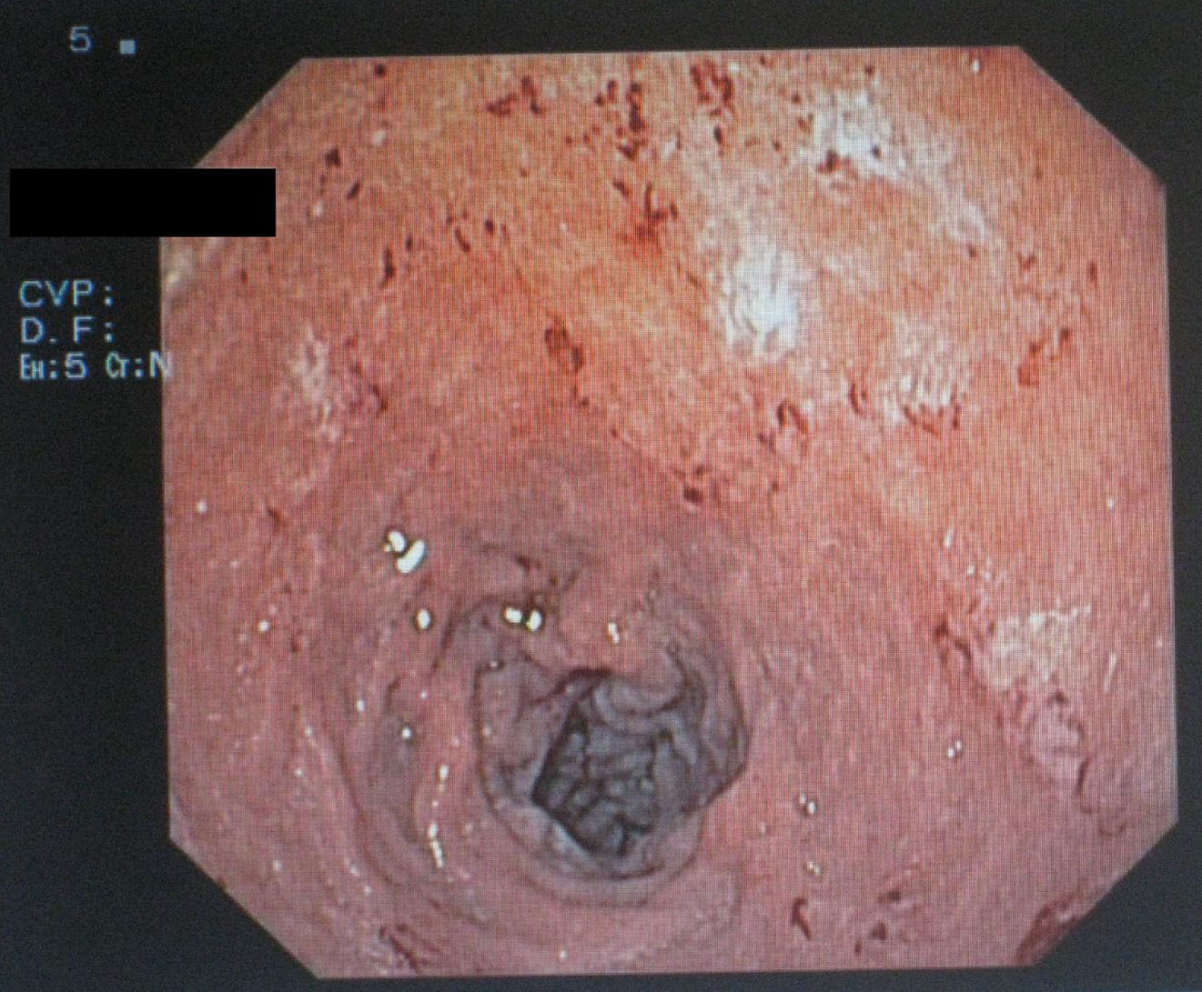
**Hemorrhagic focus at bulbus Gastroduodenoscopic view**.

**Figure 2:**
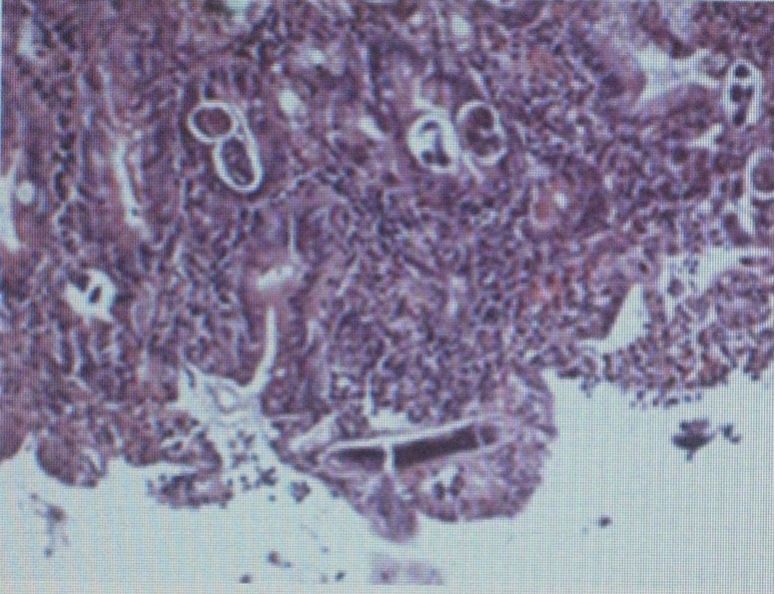
***Strongyloides *larvae are shown in gastric gland and duodenal crypts**.

**Figure 3:**
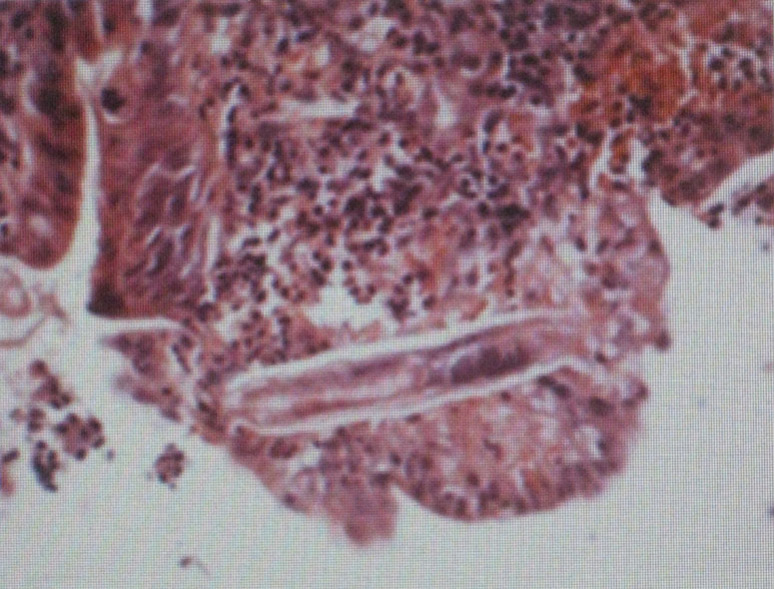
***Strongyloides *larvae are shown in gastric gland and duodenal crypts**.

In our patient, oesophagogastroduodenoscopy performed and showed hemorrhagic focus at bulbus (Figure 1). Gastric biopsy obtained and showed evidence of *S.Stercoralis *infection (Figures 2 and 3). Microscopically, chronic gastritis and scattered eosinophils were observed. Also, histological examination showed numerous larvae in gastric glands and duodenal crypts.

**Figure 4:**
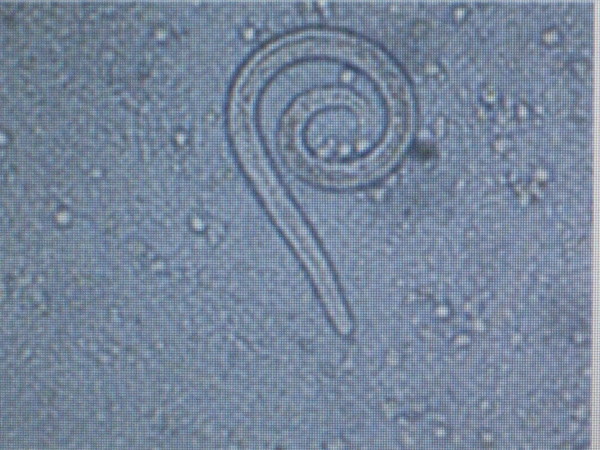
**Direct microscopic examination of sputum**.

**Figure 5:**
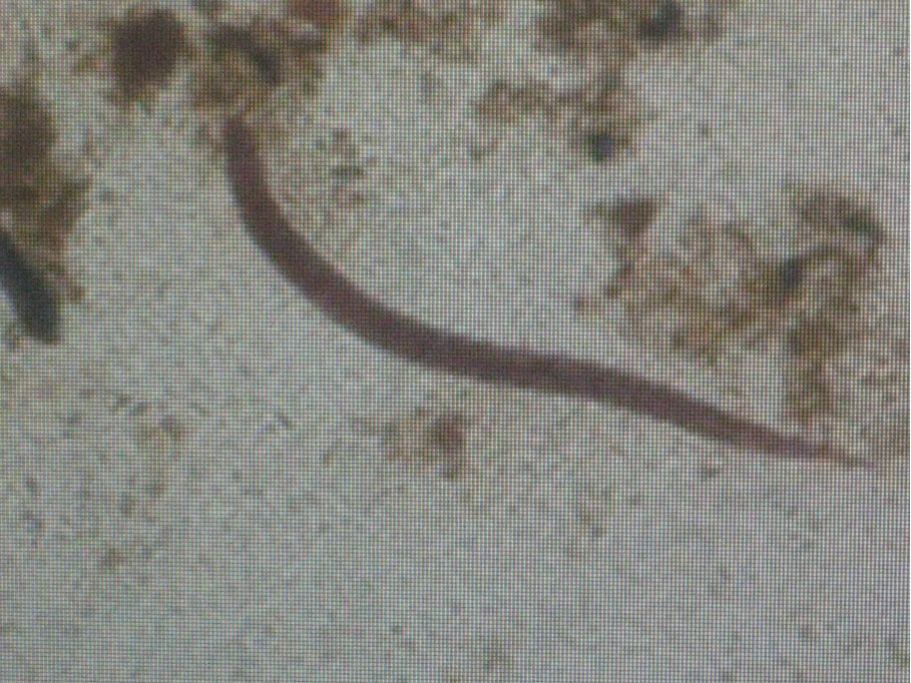
**Lugol strained gaita sample, the parasite larvae feature**.

Stool and sputum parasitological examination were taken. They were also all positive for *S.stercoralis *larvae. The samples' microscopic features are shown at Figures 4 and 5, respectively.

Chest radiography had no pathologic finding. Blood microscopic examination was negative for larvae. HIV test was negative. Albendazole 400 mg/day was started. The steroid therapy ceased by decreasing the dose. At the 6th day of therapy, sputum and stool samples were negative for alive *S.stercoralis *larvae. The therapy stopped at 15th day.

2 days after cessation of therapy, sputum sample became positive again for larvae. Ivermectin planned; but, as it was not found in our country, albendazole therapy continued till the drug was provided. At the 23 th day of albendazole, stool and sputum were negative. After the ivermectin obtained, patient was treated with oral ivermectin 200 μg once daily for 3 days. On her outpatient control at 15th day, stool and sputum samples were all negative for parasite.

## Discussion and Review of Literature

*Strongyloides stercoralis *is an intestinal nematode that is widely distributed throughout the tropics and subtropics[1]. Definitive diagnosis of strongyloidiasis usually depends on the demonstration of *S. Stercoralis *larvae in the feces or duodenal fluid. However, in a majority of uncomplicated cases, the intestinal worm load is low and the larval output is minimal. A single stool examination thus fails to detect larvae in up to 70% of cases [4]. *Strongyloides stercoralis *is a unique parasite that can cause a mortal disease years after the exposure. Lam et al. [7] carried out a retrospective study and searched seven patients characteristic features. All the patients left the endemic area more than 20 years ago.

When immunosupression develops, autoinfection accelerates and hyperinfection occurs. Hyperinfection has been associated with large numbers of parasites. The larvae do not spread out in the normal migration pattern but restricted to GI tract and lungs, whereas disseminated infection results in spreading to any organ. Multiple organs are affected including lungs, liver, heart, kidneys and central nervous system. Major complaints were fever, abdominal pain, diarrhoea, abdominal distension, weight loss, vomiting, cough, anemia, hemoptysis[7-11].

Under some conditions associated with immunocompromise, this autoinfective cycle can become amplified into a potentially fatal hyperinfection syndrome, characterized by increased numbers of infective filariform larvae in stool and sputum and clinical manifestations of the increased parasite burden and migration, such as gastrointestinal bleeding and respiratory distress. *S. stercoralis *hyperinfection is often accompanied by sepsis or meningitis with enteric organisms. Glucocorticoid treatment and human T-lymphotropic virus type 1 infection are the two conditions most specifically associated with triggering hyperinfection, but cases have been reported in association with hematologic malignancy, malnutrition, and AIDS [5].

In most cases, in the literature, primary complaints were occasionally evaluated as different diagnosis than *strongyloides*; but, after that duodenal biopsy, bronchoalveolar lavage, stool and sputum samples showed the parasite.

In the literature, there were cases with marked gastrointestinal symptoms [7-10]. Lam et al. [7] stated that 4 of their 7 patients had intestinal ileus clinic with vomiting whom required nazogastric decompression. Das et al. [8] presented a farmer with seropositive RA with chronic diarrhea. Krishnamurthy et al. [9] also reported a RA case with vomiting and abdominal discomfort. Culha et al. [10] marked a case with diarrhea which differs from the other studies as the patient had no immunosupressive status. Diarrhea and abdominal distension were not present at our patient, and also she had constipation story. Segarra-Newnham [11] reviewed the literature for *S.stercoralis *cases and over 50 of these cases hyperinfection and disseminated infection were found. In this study, the most common risk factor was found to be the steroid usage (64%). Our patient also has been on low dose steroid therapy.

In our case, the patient's gastrointestinal complaint was dyspepsia and vomiting. She had complaints about coughing and shortness of breath; but, expressed that it was present till her asthma was developed. We first evaluated her respiratory symptoms to her primary disease and did not think of a parasitic disease till we took the pathology result. After *S.stercoralis *identified in duodenal biopsy samples, we took recurrent stool and sputum samples, and all were positive for the parasite. After that, albendazole therapy was started. When we started the therapy, the patient's hyponatremia and mild anemia was improved firstly.

## Conclusion

*S.stercoralis *can cause mortal diseases in patients. The parasite is unique among the parasitic nematodes because of its ability to multiply within the human host even after many decades, with the potential to cause life-threatening disease in immunocompromised patients. Immunosupression frequently cause disseminated infection and the disease control rates unfortunately are not in the desirable ratio. In immunosupressed patients, to evaluate *S.stercoralis *infection might help for the survival of the patient and constitute the exact therapy.

## Consent

Written informed consent was obtained from the patientfor publication of this case report after the patient expired. A copy of the written consent is available for review from the Editor-in-Chief of this journal.

## Competing interests

The authors declare that they have no competing interests.

## Authors' contributions

The authors conceived of this case report, gathered the source material, and drafted the manuscript. All authors read and approved the final manuscript.
